# Diterpenoid Caesalmin C Delays Aβ-Induced Paralysis Symptoms via the DAF-16 Pathway in *Caenorhabditis elegans*

**DOI:** 10.3390/ijms23126871

**Published:** 2022-06-20

**Authors:** Zong-Ping Zhang, Xue Bai, Wen-Bo Cui, Xiao-Han Chen, Xu Liu, De-Juan Zhi, Zhan-Xin Zhang, Dong-Qing Fei, Dong-Sheng Wang

**Affiliations:** 1 School of Pharmacy, Lanzhou University, Lanzhou 730000, China; zhangzp19@lzu.edu.cn (Z.-P.Z.); baix2020@lzu.edu.cn (X.B.); cuiwb19@lzu.edu.cn (W.-B.C.); chenxh2019@lzu.edu.cn (X.-H.C.); liuxu19@lzu.edu.cn (X.L.); zhidej@lzu.edu.cn (D.-J.Z.); zhangzhx@lzu.edu.cn (Z.-X.Z.); 2 State Key Laboratory of Applied Organic Chemistry, Lanzhou University, Lanzhou 730000, China

**Keywords:** caesalmin C, Alzheimer’s disease, *Caenorhabditis elegans*, amyloid β-protein, DAF-16

## Abstract

Alzheimer’s disease (AD) is the most prevalent neurodegenerative disease in the world. However, there is no effective drug to cure it. Caesalmin C is a cassane-type diterpenoid abundant in *Caesalpinia bonduc* (Linn.) Roxb. In this study, we investigated the effect of caesalmin C on Aβ-induced toxicity and possible mechanisms in the transgenic *Caenorhabditis elegans* AD model. Our results showed that caesalmin C significantly alleviated the Aβ-induced paralysis phenotype in transgenic CL4176 strain *C. elegans*. Caesalmin C dramatically reduced the content of Aβ monomers, oligomers, and deposited spots in AD *C. elegans*. In addition, mRNA levels of *sod-3*, *gst-4*, and *rpt-3* were up-regulated, and mRNA levels of *ace-1* were down-regulated in nematodes treated with caesalmin C. The results of the RNAi assay showed that the inhibitory effect of caesalmin C on the nematode paralysis phenotype required the DAF-16 signaling pathway, but not SKN-1 and HSF-1. Further evidence suggested that caesalmin C may also have the effect of inhibiting acetylcholinesterase (AchE) and upregulating proteasome activity. These findings suggest that caesalmin C delays the progression of AD in *C. elegans* via the DAF-16 signaling pathway and that it could be developed into a promising medication to treat AD.

## 1. Introduction

Alzheimer’s disease (AD) is a progressive, irreversible neurodegenerative disease. It is clinically characterized by cognitive decline, memory loss, and executive function impairment. AD accounts for about 55–75% of dementia, and the incidence of AD increased dramatically along with the aging of society’s population [1]. It is expected that the total number of AD patients will reach 78 million by 2030 and 139 million by 2050, which will bring a heavy burden to both society and patients’ families [2]. Therefore, effective prevention and treatment of AD became an urgent research topic worldwide. The pathology of AD is characterized by senile plaques (SP) formed by extracellular amyloid-β (Aβ), intracellular neurofibrillary tangles (NFT) composed of hyperphosphorylated Tau protein, and synaptic loss [3]. According to the Aβ hypothesis, the excessive production and aggregation of Aβ in the brain leads to ion channel blockage, dysregulation of calcium homeostasis, increased mitochondrial oxidative stress, and induction of inflammatory cascade response that ultimately leads to neuronal cell death [4]. Therefore, inhibition of Aβ toxicity can be effective in the prevention and treatment of AD.

Currently, the main clinical anti-AD drugs are AchE inhibitors (donepezil, rivastigmine, and galanthamine) and N-methyl-d-aspartate (NMDA) receptor antagonists (memantine), all of which can only relieve the symptoms but not completely cure AD, and most of them have serious side effects [5]. A number of natural terpenoids were found to be promising multi-targeted agents that may help to modulate several changes associated with a disease, thus helping to overcome resistance deficits associated with specific target drugs and reduce their side effects [6]. In fact, several natural terpenoids were found to possess anti-AD activity. For example, the triterpenoid betulinic acid is a potent proteasome activator and also has antioxidant, anti-inflammatory, and modulating hippocampal neurochemistry activities that improve memory impairment and neurotransmitter deficits in STZ-induced AD rats [7,8]. The diterpenoid triptolide can restore neural damage caused by Aβ_25–35_ aggregation through autophagy pathway and through inhibition of oxidative stress [9]. Cornel iridoid glycoside inhibits Tau protein hyperphosphorylation by activating P13K/AKT and PP2A signaling pathways together to inhibit GSK-3β activity [10]. In addition, ginsenosides, ginkgolides, oleanolic acid, and tenuifolin can also improve AD-related pathological symptoms [11]. Therefore, natural terpenoids may be ideal lead compounds for AD, a disease with a complex etiology.

*Caesalpinia bonduc* (Linn.) Roxb., (family: Leguminosae, genus: Caesalpinia) is widely distributed in tropical and subtropical regions. As a traditional folk medicine, it is often used to treat fever, dysentery, and asthma [12,13]. Phytochemical analysis showed that the plant contains mainly polyphenols, flavonoids, saponins, and terpenoids. The plant has mainly anti-inflammatory, antioxidant, antiviral, and hypoglycemic activities [14]. Caesalmin C, one of the characteristic components of *C. bonduc*, was found to have good anti-Para3 virus activity [15]. In addition, caesalmin C exhibited significant anti-inflammatory activity and inhibited Nitric Oxide (NO) production up to 71.2% in LPS-induced RAW264.7 cells [16].

*Caenorhabditis elegans* (*C. elegans*) provides a convenient and effective pathological model for assessing the efficacy of anti-AD drug candidates and their mechanism of action against Aβ toxicity. In the present study, we evaluated for the first time the anti-AD activity of caesalmin C using transgenic CL4176 nematodes and found that the compound significantly delayed the progression of nematode paralysis. We also revealed a mechanism for the protective effect of caesalmin C, that is, the reduction of Aβ toxicity through DAF-16 pathways. These results will provide data to explore the potential medicinal value of caesalmin C in the prevention of AD.

## 2. Results

### 2.1. Caesalmin C Alleviated the Symptoms of Aβ Toxicity-Induced Paralysis

**Figure 1:**
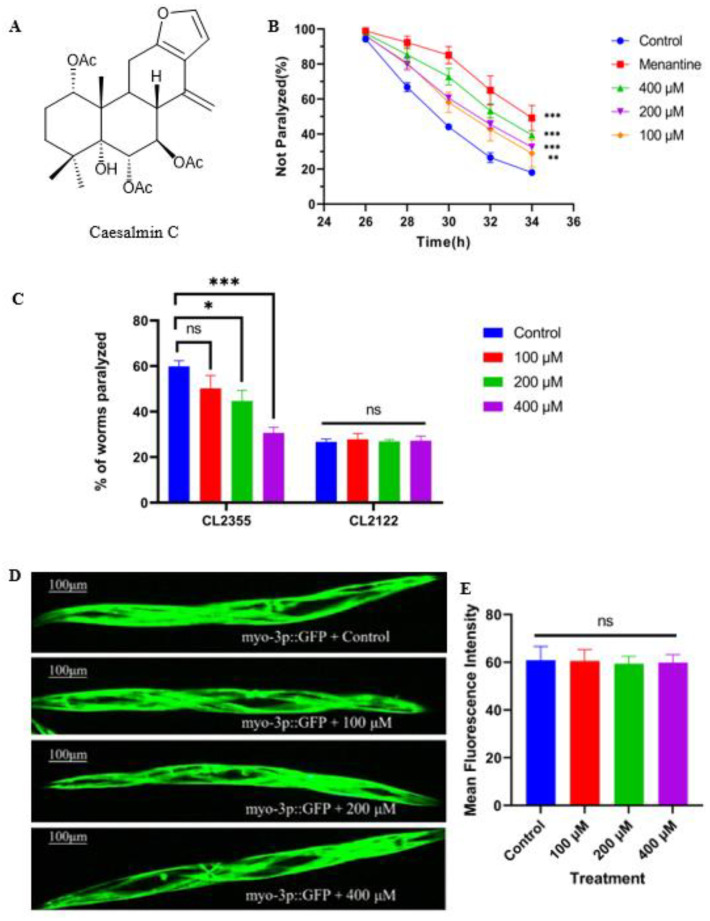
Caesalmin C alleviates paralysis symptoms in transgenic *C. elegans* expressing Aβ. (**A**) Chemical structure of caesalmin C. (**B**) The curves of the not paralyzed rate in each group. Transgenic CL4176 worms were treated with 100, 200, and 400 µM of caesalmin C, respectively. The 100 μM memantine was used as a positive control. (**C**) The effect of caesalmin C on hypersensibility to exogenous 5-HT in transgenic CL2355 worms. (**D**) The fluorescence pictures of CL2179 worms after treatment with different concentrations of caesalmin C. (**E**) The fluorescence intensity of GFP in CL2179 worms in each treated group. In all of the above, 0.1% (*v*/*v*) DMSO was used as control. Data are expressed as mean ± SD. *p* < 0.05 was considered statistically significant. The asterisk indicates significant difference compared to the respective control (ns *p* > 0.05, * *p* < 0.05, ** *p* < 0.01, *** *p* < 0.001).

The transgenic CL4176 nematodes expressed the human-derived Aβ_1–42_ gene under the *myo-3* promoter in body wall muscle cells, which were induced at 25 °C, resulted in massive expression of Aβ_1–42_ protein, leading to paralysis symptoms in nematodes. The chemical structure of caesalmin C is shown in the figure (Figure 1A). We evaluated the anti-AD activity of caesalmin C with this strain of nematodes and the result showed that all three concentrations of caesalmin C significantly inhibited nematodes paralysis. The inhibition rate was up to 39.4% at a concentration of 400 µM (Figure 1B). Next, we evaluated the effect of caesalmin C on Aβ toxicity in nematode neurons using the CL2355 strain. The transgenic CL2355 nematodes showed deficits in chemotaxis, associative learning, and thrashing in liquid, and when they were induced at 25 °C, Aβ_1–42_ peptides were abundantly expressed in neuronal cells, leading to a hypersensitive response to exogenous 5-HT and eventually to a rigid state. CL2122 nematodes were used as a transgenic background control for CL2355. The results showed that caesalmin C significantly inhibited the sensitivity of AD nematodes to exogenous serotonin, and that the 400 μM group restored the nematodes to normal levels (Figure 1C). The CL2179 strain of nematodes imports only GFP under the *myo-3* promoter, so we used this strain to explore whether caesalmin C inhibits all exogenous proteins. As expected, there was no significant difference in fluorescence intensity between the three caesalmin C concentration treatment groups and the blank group, demonstrating that this diterpenoid acts specifically on the Aβ_1–42_ protein (Figure 1D,E).

### 2.2. Caesalmin C Inhibited Aβ Aggregation

**Figure 2:**
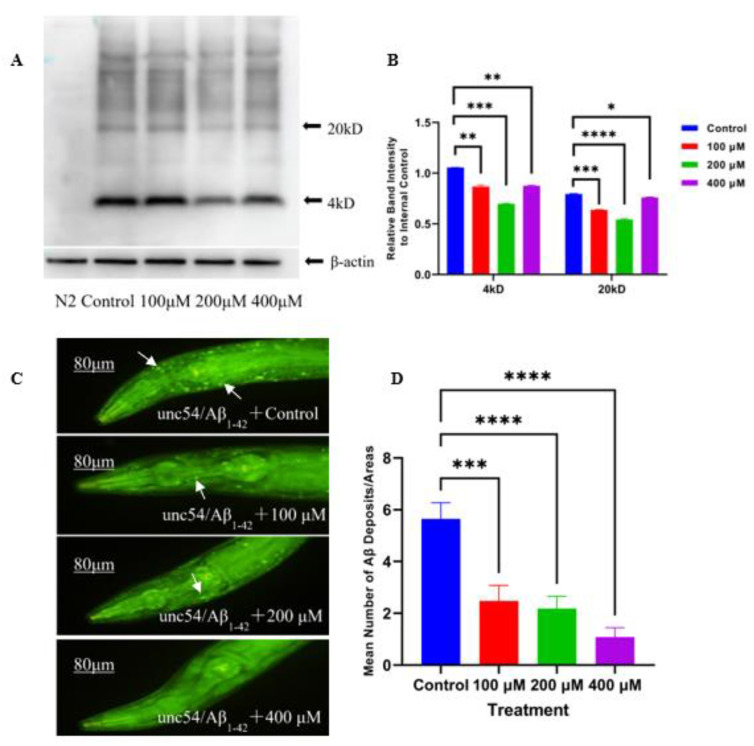
Caesalmin C reduced Aβ monomers, oligomers and deposits in worms. (**A**) WB assay of Aβ expression level in transgenic CL4176 worms of each treated group. Wild-type (N2) worms were used as a transgenic control and did not express Aβ_1–42._ β-actin was used as an internal reference. (**B**) The quantitative analysis of Aβ monomers and Aβ oligomers in each treated group. (**C**) Aβ deposits in the pharynx of transgenic CL2006 worms in each treatment group after ThS staining. (**D**) The quantitative analysis of Aβ deposits in each treated group. In all of the above, worms were treated with 100, 200, and 400 µM of caesalmin C, respectively, 0.1% (*v*/*v*) DMSO was used as control. Data are expressed as mean ± SD. The asterisk indicates significant difference compared to the respective control (* *p* < 0.05, ** *p* < 0.01, *** *p* < 0.001, **** *p* < 0.0001).

The Aβ species are broadly classified into three types: monomers, oligomers, and fibrils. Among them, oligomers are considered to be more toxic than monomers and fibrils [17]. In this research, the effect of caesalmin C on the expression levels of Aβ monomers (4 kDa) and oligomers (20 kDa) was examined using Western blot (WB) technique. The results showed that all three concentrations of caesalmin C could effectively decrease the content of Aβ monomers and oligomers. Among them, the concentration of 200 μM could reduce the Aβ protein content of 4 kDa and 20 kDa by 33.9% and 31.6%, respectively (Figure 2A,B). In addition, staining of Aβ deposits in transgenic CL2006 nematodes using thioflavine S (ThS) showed that all three concentrations of caesalmin C reduced Aβ deposits by more than 55.2% (Figure 2C,D). These evidenced that caesalmin C significantly inhibited the formation of Aβ aggregates to lower Aβ toxicity.

### 2.3. DAF-16 Signaling Pathway Played an Important Role in the Alleviation of Nematode Paralysis by Caesalmin C

Both NRF2/SKN-1 and FOXO/DAF-16 are key components downstream of the insulin signaling pathway and play important roles in resisting aging and oxidative stress, regulating metabolism, and improving stress tolerance [18,19,20]. Heat shock factor 1 (HSF-1) is a major regulator of the heat shock response (HSR). When cells are subjected to high temperature, oxidative stress, or other harsh environmental stimuli, HSF-1 rapidly transitions to an active form that promotes the expression of heat shock proteins (HSPs) and ensures the refolding or degradation of misfolded proteins in stressed cells [21].

**Figure 3:**
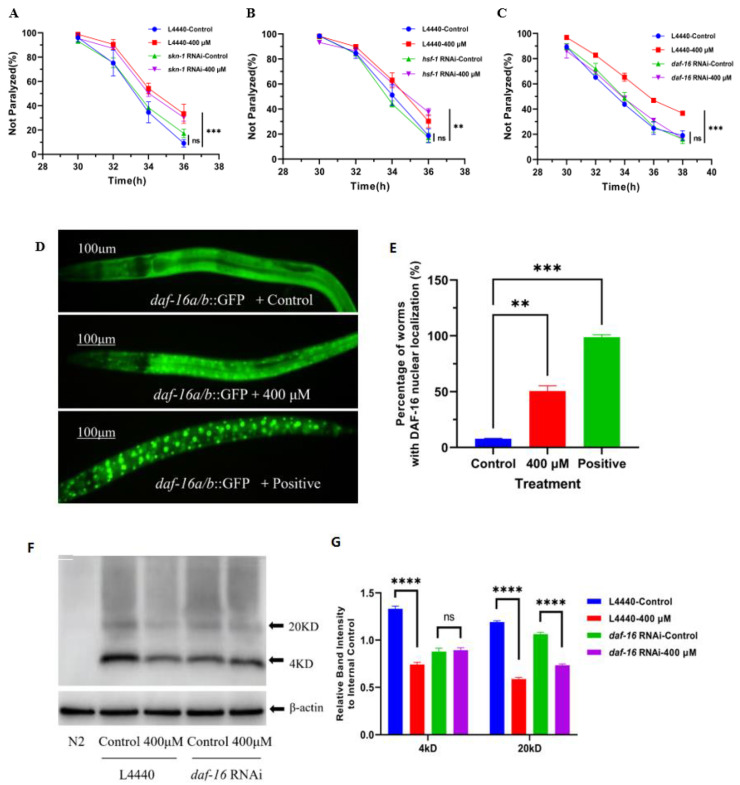
Caesalmin C alleviates Aβ-induced toxicity in nematodes mediated by DAF-16 signal pathway. (**A**) The curves of the not paralyzed rate in transgenic CL4176 worms after treatment with or without *skn-1* RNAi. (**B**) The curves of the not paralyzed rate in transgenic CL4176 worms after treatment with or without *hsf-1* RNAi. (**C**) The curves of the not paralyzed rate in transgenic CL4176 worms after treatment with or without *daf-16* RNAi. (**D**) Caesalmin C at 400 µM induced DAF-16 from the cytoplasm into the nucleus in TJ356 worms. Worms were incubated at 37 °C for 30 min as the positive control. (**E**) The quantitative analysis of the worms with DAF-16 nuclear localization in each treated group. (**F**) WB detected Aβ expression levels in each group of CL4176 worms. The *daf-16* expression in CL4176 worms was knocked down by RNAi and L4440 was used as an empty vector control. (**G**) The quantitative analysis on Aβ monomers and Aβ oligomers in each treated group. In all of the above, worms were treated with 400 µM of caesalmin C, and 0.1% (*v*/*v*) DMSO was used as control. Data are expressed as mean ± SD. The asterisk indicates significant difference compared to the respective control (ns *p* > 0.05, ** *p* < 0.01, *** *p* < 0.001, **** *p* < 0.0001).

The expressions of *skn-1*, *daf-16,* and *hsf-1* in CL4176 nematodes were knocked down by RNAi to test whether the effect of caesalmin C in delaying nematode paralysis would be changed. The results showed that there was no obvious change in the nematodes paralysis delaying effect of caesalmin C after the reduction of *skn-1* and *hsf-1* expressions, indicating that caesalmin C does not require the involvement of SKN-1 and HSF-1 signaling pathways to function (Figure 3A,B). However, the nematode paralysis inhibitory effect of caesalmin C was abolished after the knockdown of *daf-16* expression (Figure 3C). Next, the results of nuclear localization experiments in DAF-16 showed that 400 μM caesalmin C could effectively promote the movement of DAF-16 in the cytoplasm to the nucleus (Figure 3D,E). In addition, WB experiments were performed to test whether the inhibitory effect of caesalmin C on Aβ production was affected by knocking down *daf-16* expression in nematodes. The results showed that the effect of 400 μM caesalmin C in reducing Aβ monomers was lost and the effect of reducing Aβ oligomers was reduced from 50.7% to 30.9%, but did not disappear completely (Figure 3F,G). In summary, the DAF-16 signaling pathway is key, but not the only mechanism by which caesalmin C exerts its effect.

### 2.4. Caesalmin C Promoted the Expression of Sod-3 and Gst-4 in C. elegans

**Figure 4:**
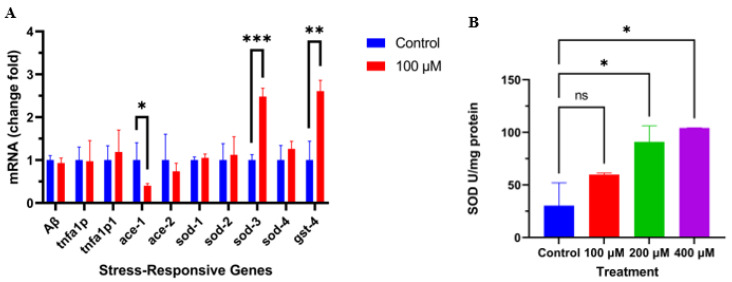
Caesalmin C improves the resistance of *C*. *elegans* to oxidative stress. (**A**) The effect of caesalmin C on the expression level of stress-responsive genes in CL4176 worms. β-actin was used as an internal reference. (**B**) The effect of caesalmin C on the SOD activity in CL4176 worms. In all of the above, 0.1% (*v*/*v*) DMSO was used as control. Data are expressed as mean ± SD. The asterisk indicates significant difference compared to the respective control (ns *p* > 0.05, * *p* < 0.05, ** *p* < 0.01, *** *p* < 0.001).

Based on the previous results, we used qRT-PCR to detect the effect of caesalmin C on the expression of genes downstream of the DAF-16 signaling pathway. The results showed that caesalmin C dramatically promoted the expressions of *sod-3* and *gst-4* mRNA in CL4176 nematodes (Figure 4A). Then, we measured the changes of SOD activity in CL4176 nematodes after caesalmin C treatment by the NBT method. The results showed that caesalmin C could significantly increase the SOD activity in nematodes (Figure 4B). At the same time, we also examined the mRNA expression levels of *ace-1*, *ace-2*, *TNFA1P*, *TNFA1P1,* and *Aβ*. The results showed that the expression level of *ace-1* was obviously decreased (Figure 4A). This suggests that caesalmin C may be a potent cholinesterase inhibitor.

### 2.5. Caesalmin C Promotes the Expression of Rpt-3p::GFP in C. elegans

**Figure 5:**
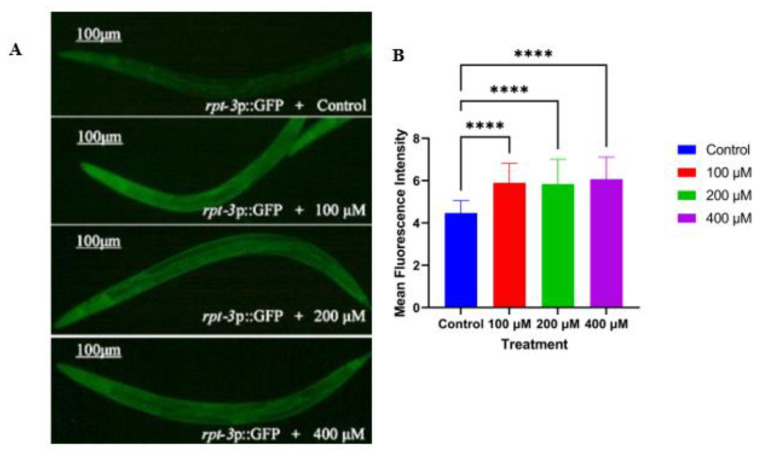
Caesalmin C promotes the expression of *rpt-3* in GR2183 *C. elegans*. (**A**) The fluorescence pictures of worms in each treated group. (**B**) The quantitative analysis of worm fluorescence intensity in each treatment group. The 0.1% DMSO was used as control and the 100, 200, and 400 μM were the final concentrations of caesalmin C in NGM, respectively. Data are expressed as mean ± SD. The asterisk indicates significant difference compared to the respective control (**** *p* < 0.0001).

The ubiquitin-proteasome system (UPS) is one of the major degradation mechanisms of abnormal or misfolded proteins. Any disturbance of the UPS leads to the accumulation of abnormal proteins, which aggravates AD [22]. GR2183 *C. elegans* was used to detect the expression level of the proteasome-related factor *rpt-3*, and the intensity of green fluorescence in nematodes was enhanced when the compound promoted *rpt-3* expression. The results showed that all three concentrations of caesalmin C significantly upregulated the expression of *rpt-3.* It was suggested that caesalmin C may accelerate the clearance of abnormal proteins in AD nematodes by upregulating UPS activity (Figure 5A,B).

## 3. Discussion

DAF-16 is a homologous protein of mammalian FOXO, which plays a key role in normal lifespan and stress resistance in mice, drosophilas, and *C. elegans* [23,24]. It was found that DAF-16 is involved in the regulation of development, reproduction, metabolism, and apoptosis, in addition to its role in resistance and longevity [25]. DAF-16 is a central regulator of multiple signaling pathways that are involved in nutrient uptake, cell proliferation, and development, and DAF-16 integrates this information to initiate stress response to increase protein balances, improve stress resistance, and maintain cellular homeostasis [26]. The downstream genes of DAF-16, *hsp-16* and *hsp-12.6*, are required for heat resistance in nematodes, and the antioxidant genes, *sod-3* and *ctl-1*, are required to enhance the resistance to oxidative stress in *daf-2* mutants [27]. Some studies have shown that the DAF-16 signaling pathway can regulate the formation of highly toxic small molecular weight Aβ protein aggregates into low toxicity, high molecular weight aggregates, which also have a role in resistance to Aβ-induced toxicity [28]. Our results indicated that the protective effect of caesalmin C requires DAF-16. Further studies showed that caesalmin C at least partially induced the translocation of DAF-16 from the cytoplasm to the nucleus. Furthermore, after knocking down DAF-16 expression in nematodes with RNAi, the effect of caesalmin C in reducing Aβ oligomers content was significantly reduced. This evidence, again, demonstrates that the DAF-16 signaling pathway contributes to the resistance to Aβ toxicity. However, we still cannot elucidate whether DAF-16 promotes the degradation of Aβ aggregates or inhibits the formation of Aβ aggregates or both.

Notably, *gst-4* is a recognized target gene of *skn-1*, and *sod-3* is a recognized target gene of *daf-16*. However, it was also found that they were cross-linked and not completely independent pathways, and *gst-4* was also regulated by *daf-16* [29,30]. This reasonably explained that caesalmin C significantly promoted the expression of *gst-4* but did not act on the SKN-1 signaling pathway. Clinical studies have shown that oxidative stress occurs prior to neuronal and tissue damage in the brain and is an important factor in the development of AD [31]. Excessive production of ROS in the brain leads to oxidative deterioration of lipids, proteins, and nucleic acids in neurons, exacerbating the disease [32]. GST-4 is involved in phase II detoxification and SOD-3 scavenges O^2−^ radical, both of which reduce oxidative damage and improve stress resistance in *C. elegans* [33,34]. In conclusion, the expressions of *gst-4* and *sod-3* are beneficial in resisting the toxicity of Aβ.

The cholinergic dysfunction hypothesis suggests that memory deficits in AD patients are positively correlated with cholinergic impairments and that increasing the intrasynaptic acetylcholine (ACh) content significantly improves cognitive performance and memory levels [35]. According to previous reports, AChE interacts with Aβ and promotes amyloid fibril formation through a hydrophobic environment at the enzyme’s periphery [36]. Kinetic analyses indicate that the hydrophobic sequence of 35 peptides in AChE accelerates the formation of Aβ and can insert into growing Aβ protofibrils [37]. This evidence suggested the involvement of AChE in the pathogenesis of AD. Currently, the clinical treatment of AD is also dominated by AchE inhibitors. In the present work, the results of qRT-PCR experiments showed that caesalmin C significantly reduced the expression of *ace-1*, suggesting that caesalmin C may exert its anti-Aβ-induced toxicity by inhibiting the activity of AchE.

There is increasing evidence that the generation and untimely clearance of large amounts of misfolded proteins play an important role in the pathogenesis of AD [38]. The ubiquitin-proteasome system (UPS) and autophagy pathways are the main degradation mechanisms of intracellular proteins. Oleuropein and quercetin were found to exert neuroprotective effects through upregulation of proteasome activity and induction of autophagy [39,40]. Fucoidan was reported to increase proteasome activity and promote Aβ protein hydrolysis to alleviate neurotoxicity in transgenic AD *C. elegans* [41]. These studies suggested that UPS and autophagy may provide effective therapeutic targets for the treatment of AD. The 26S proteasome is a large ATP-dependent protein hydrolysis complex that usually catalyzes most abnormal protein degradation after attachment to the ubiquitin chain. RPT-3 is a key factor in maintaining the stability of the 26S proteasome complex and exerting its hydrolytic effect [42,43]. In the current study, combining the results of WB experiments and fluorescence detection experiments, it was shown that caesalmin C could significantly reduce the content of Aβ oligomers and promote the expression of *rpt-3* in nematodes. This evidence suggests that caesalmin C might also degrade Aβ toxic proteins by activating UPS, but the detailed mechanism of action needs to be further investigated.

Previously, caesalmin C was reported as a potent antiviral and anti-inflammatory agent in vitro [15,16], but little information is available on the antioxidant properties of caesalmin C and its possible role in alleviating Aβ-induced cytotoxicity in *C. elegans.* In this research, the beneficial effects of caesalmin C on such strains were investigated, and the mechanisms involved were explored at the molecular level. Collectively, these results provide data to support the prevention and treatment of AD by diterpenoids and offer new ideas for the development of anti-AD drugs and the utilization of *C. bonduc.*

## 4. Materials and Methods

### 4.1. Chemicals and Treatment

5-HT was purchased from Alfa Aesar Company (Shanghai, China). Thioflavin S was purchased from Sigma-Aldrich (St Louis, MO, USA). Dimethyl sulfoxide (DMSO) was purchased from Solarbio Company (Beijing, China). Caesalmin C was extracted, isolated, and purified from the seeds of *C. bonduc* in our laboratory. The seeds of *C. bonduc* were purchased in April 2014 from Anguo Traditional Chinese Medicine Market in Hebei Province, China, and identified by Dr. Jian-Yin Li of School of Pharmacy, Lanzhou University. A voucher specimen (No. 20140418CB) was deposited at School of Pharmacy, Lanzhou University, China. The spectral information of caesalmin C is presented in the Supplementary Material. The caesalmin C was dissolved in DMSO, mixed with NGM, and poured into Petri plates. Under all conditions, the final concentrations of DMSO were maintained at 0.1% (*v*/*v*).

### 4.2. C. elegans Strains and Handling Conditions

*C. elegans* strains used in this study are listed as follows: wild-type N2; CL4176, dvIs27 [myo-3p::A-Beta (1–42)::let-851 3′UTR) + rol-6(su1006)]; CL2006, dvIs2 [pCL12 (unc-54/human Aβ peptide1−42minigene) + pRF4]; CL2355, dvIs50 [pCL45 (snb-1::Abeta 1–42::3′ UTR(long) + mtl-2::GFP]; CL2122, dvIs15 [(pPD30.38) unc-54(vector) + (pCL26) mtl-2::GFP]; CL2179, dvIs179 [myo-3p::GFP::3′ UTR(long) + rol-6(su1006)]; TJ356, zIs356 [daf-16p::daf-16a/b::GFP + rol-6]; CL2166, dvIs19 [(pAF15) gst-4p::GFP::NLS] III; CF1553, muIs84 [(pAD76) sod-3p::GFP + rol-6(su1006)]; GR2183, mgIs72 [rpt-3p::GFP + dpy-5(+)] II. All *C. elegans* strains and *Escherichia coli* OP50 were purchased from the Caenorhabditis Genetics Center (CGC; University of Minnesota, St. Paul, MN, USA). Worms of CL4176, CL2355, CL2122, and CL2179 were cultured on nematode growth medium (NGM) plates seeded with *E. coli* OP50 as food resources in an incubator at 15 °C; the other strains were grown at 20 °C. To harvest age-synchronized animals, pregnant adults were treated with hypochlorite and eggs were washed three times with M9 buffer.

### 4.3. Paralysis Assays

CL4176 transgenic nematodes regulate Aβ peptide expression by transferring an Aβ_1–42_ gene containing a 3′UTR sequence under the body wall muscle myo-3 promoter. This strain contains the temperature-sensitive gene, smg-1. At permissible temperatures, the transcription product of the 3′UTR was degraded and the Aβ peptide was barely expressed. When the temperature rose to 25 °C, the smg-1 gene was functionally inactivated, the Aβ transcript was stably expressed, and a large amount of Aβ protein accumulated in the nematode body wall muscle, resulting in nematodes showing paralytic symptoms [39].

Synchronized eggs were incubated at 15 °C for 30 h and then transferred to fresh NGM plates containing caesalmin C 100 µM, 200 µM, and 400 µM, respectively. A mixture of 0.1% DMSO and 0.3% PEG400 was used as solvent control. When the nematodes reached L3-stage, the incubation temperature was changed from 15 °C to 25 °C. After 28 h, paralyzed individuals were counted every 2 h. When lightly touched with a platinum wire ring, the nematodes were immobile. If only the head could move, it was considered paralyzed. Three independent biological replicates were performed in this experiment.

### 4.4. Exogenous Serotonin Sensitivity Assay

CL2355 transgenic nematodes also contained the smg-1 temperature monitoring system, and the human-derived Aβ_1–42_ gene was transferred under the snb-1 promoter in neurons. After treatment at 25 °C, smg-1 was functionally inactivated and Aβ protein was expressed in large numbers in nematode neurons, resulting in a substantial reduction in tolerance to exogenous 5-HT in this strain of nematodes, which eventually exhibited a state of rigidity [40].

Synchronized L1 larvae were transferred to fresh NGM plates, with or without caesalmin C amounts of 100 µM, 200 µM, and 400 µM, treated at 15 °C for 65 h, then transferred into a 25 °C incubator for 30 h. The nematodes were collected in M9 buffer, and the number of paralytic nematodes was counted on 96-well plates treated with 5 mg/mL of serotonin for 5 min. CL2122 was used as a transgenic control, and this experiment was repeated at least three times.

### 4.5. Effect of Caesalmin C on Exogenous Protein Expression in C. elegans

As a control strain of CL4176 nematodes, CL2179 nematodes were transferred only to green fluorescent protein (GFP) under myo-3 promoter. This strain of nematodes expresses GFP in large amounts in muscle cells when induced at 25 °C, resulting in a strong green fluorescence around the body. Synchronized eggs of CL2179 were incubated at 15 °C for 30 h, and then seeded on fresh NGM plates with or without caesalmin C in amounts of 100 µM, 200 µM, and 400 µM. They were treated at 15 °C for 67 h, followed by 25 °C for 30 h. The nematodes were washed with M9 buffer and collected. The nematodes were fixed in the center of the slide with 20 mM sodium azide, covered with a coverslip, and the overall fluorescence intensity of the nematodes was observed and recorded under a fluorescence microscope (DS-Ri2; Nikon, Japan). At least 30 nematodes were recorded in each treatment group. The fluorescence intensity of the whole body of the nematode was quantified with ImageJ software.

### 4.6. Fluorescence Staining of Aβ Deposits Assay

Transgenic CL2006 strain nematodes expressed human-derived Aβ_1–42_ protein slowly in muscle cells when cultured at 20 °C, resulting in progressive nematode paralysis. Synchronized eggs were incubated at 20 °C for 30 h, and then transferred to fresh NGM plates with or without caesalmin C amounts of 100 µM, 200 µM and 400 µM, followed by treatment at 20 °C for 6 days. The nematodes were then collected in M9 buffer and washed three times, after which 4% tissue cell fixative was added and placed in a 4 °C refrigerator for 24 h. The supernatant was removed by centrifugation and placed at 37 °C for 24 h after the addition of 125 mM Tris–HCl (pH 7.4) with 1% TritonX-100 and 5% β-mercaptoethanol. Aβ deposits were dyed with 0.125% thioflavin S (ThS) in 50% ethyl alcohol and decolored background with 50% ethyl alcohol. Fluorescence images were taken from the same parameters under a fluorescence microscope (DS-Ri2; Nikon, Japan). The fluorescent spots in front of the pharyngeal bulb of each individual were counted using ImageJ software. Each group contained at least 30 individuals, and the experiment was performed in three independent biological replicates.

### 4.7. RNA Extraction and Quantitative Real-Time PCR Analysis

Synchronized L1 larvae of CL4176 were transferred to fresh NGM plates with or without caesalmin C and incubated at 15 °C for 65 h, followed by induction at 25 °C for 30 h. Nematodes were collected with M9 buffer and washed three times. RNA extraction and cDNA synthesis were performed according to the manufacturer’s protocols of TRIeasyTM Total RNA Extraction Kit (Yeasen Biotechnology, Shanghai, China) and Hifair^®^ III Reverse Transcriptase Kit (Yeasen Biotechnology, Shanghai, China), respectively. Real-time PCR was performed using the Hieff^®^ qPCR SYBR Green Master Mix (Yeasen Biotechnology, Shanghai, China) and applied real-time fluorescence quantification instrument (Quant Studio 3, ABI, USA). The relative quantities of mRNA were determined using 2^–ΔΔCt^ methods and normalized against the housekeeping gene (β-actin) mRNA. The sequences of forward and reverse primers used were: CCGACATGACTCAGGATATGAAGT and CACCATGAGTCCAATGATTGCA for *Aβ*; AGTGGGCTCCTGTTCGAGAA and CCAATAGAAAATCACCATCGACAA for *ace-1*; CAATAATCAACTCATGGGCATCA and TTTTCGCGAGACGAAACGA for *ace-2*; TCCCCATACGAAACAACACA and CTCCTCCCAGCTTTTCCACAA for *TNFA1P*; CCAGAAGAATCCCCATACGA and TCCTCCTCCAACTTTTCCAAA for *TNFA1P1*; CGTAGGCGATCTAGGAAATGTG and AACAACCATAGATCGGCCAACG for *sod-1*; AGCTTTCGGCATCAACTGTC and AAGTCCAGTTGTTGCCTCAAGT for *sod-2*; TTCAAAGGAGCTGATGGACACT and AAGTGGGACCATTCCTTCCAA for *sod-3*; GTTGTCTAAGTGCTGGTGG and TTCCACATGCAAGTCGGCT for *sod-4*; GCTGAAGCCAACGACTCCAT and GACCGAATTGTTCTCCATCGA for *gst-4*; CCACGTCATCAAGGAGTCAT and GGAAGCGTAGAGGGAGAGGA for *β-actin*.

### 4.8. RNA Interference (RNAi) Assay

Transgenic CL4176 nematodes were cultured on fresh NGM plates seeded with interfering bacteria for two generations and then collected for synchronization. Respectively, the RNAi NGM plates contained 100 μg/mL ampicillin, 5 μg/mL tetracycline hydrochloride, and 1 mM isopropyl β-D-thiogalactopyranoside, and were seeded with *E. coli* HT115 cloned target gene of *hsf-1*, *daf-16*, and *skn-1*, or only empty L4440 vector. Synchronized eggs were transferred to NGM plates with or without the caesalmin C and treated at 15 °C for 48 h, followed by induction at 25 °C for 30 h. Paralytic nematodes were scored according to the method of the paralysis experiment.

### 4.9. Subcellular DAF-16 Nuclear Localization Assay

The TJ356 strain integrates DAF-16 with GFP by gamma irradiation. Normally, this strain of nematodes shows a uniform green fluorescence around the body, and if the compound activates DAF-16, the DAF-16 nuclear transcription factor translocated from the cytoplasm to the nucleus, resulting in bright aggregation sites in nematodes.

Synchronized L1 larvae of TJ356 were transferred to fresh NGM plates with or without caesalmin C and treated at 20 °C for 62 h. Afterward, nematodes were collected in M9 buffer and washed three times. The nematodes were fixed in the center of the slide with 20 mM sodium azide anesthesia, and the percentage of nematodes with nuclear translocation in each group was observed and counted under a fluorescence microscope (DS-Ri2; Nikon, Japan). At least 30 nematodes were recorded in each treatment group, and the experiment was repeated three times independently.

### 4.10. Western Blot (WB) Assay

Protein extraction was performed according to the previously disclosed method [44]. Nematodes were extracted in a lysis buffer (62 mM Tris-HCl pH 6.8, 2% SDS (*v*/*v*), 10% glycerol (*v*/*v*), 4% β-mercaptoethanol (*v*/*v*), and 1× protease inhibitor) at 100 °C for 10 min, followed by ice bath cooling for 20 min and centrifugation at 4 °C for 10 min at 14,000× *g*. The supernatant was then aspirated and added to an appropriate amount of 5× loading buffer, mixed well and boiled for 10 min at 100 °C in a metal bath, then stored at −80 °C. The membrane was blocked at room temperature with 5% milk in TBS-Tween and then incubated with primary antibody (1:2000 dilution, 6E10, Biolegend, California, USA) overnight at 4 °C. β-actin (1:5000 dilution, Affinity Biosciences, Jiangsu, China) was used as an internal reference control and goat anti-mouse HRP (1:10,000 dilution, Affinity Biosciences, Jiangsu, China) was used as a secondary antibody. Finally, images were taken from the Tanon ECL detection system (FUSION SOLO6S.EDGE, VILBER, France) and quantified by ImageJ software. The molecular weights of the Aβ monomer and oligomer were 4 kDa and 20 kDa, respectively. The relative densitometry analysis normalized to β-actin values.

### 4.11. Assay of SOD Activity in C. elegans

Nitro-blue tetrazolium (NBT) is an alkaline phosphatase substrate. The reaction between xanthine and O_2_ catalyzed by xanthine oxidase produced superoxide anion, which reduces NBT to formazan with strong absorption at 560 nm. However, SOD scavenges the superoxide anion and thus inhibits the formation of formazan, so the higher the SOD activity, the lower the absorbance value. Accordingly, the effect of the compound on SOD activity can be analyzed by comparing the absorbance values at the same tissue concentration.

Synchronized L1 larvae of CL4176 were received on fresh NGM plates with or without caesalmin C, treated at 15 °C for 67 h, and then induced at 25 °C for 30 h. The nematodes were collected with M9 buffer and snap-frozen in liquid nitrogen, and the homogenate was made by adding the appropriate amount of pre-cooled PBS solution at 4 °C for ultrasonic crushing, followed by centrifugation at 4 °C for 10 min, and the supernatant was taken as a reserve. The protein content in the supernatant of each group was determined using the BCA method. The SOD activity in the supernatant of each group was measured according to the instructions of the NBT kit (Beyotime Biotechnology, Shanghai, China). Relative absorbance values were normalized by protein concentration. Referring to the method described in the literature [45], the effect of the compound on SOD activity in nematodes was assessed by SOD activity units (Units), calculated as follows.

Percentage inhibition (%) = (Ac1 − As)/(Ac1 − Ac2) × 100

Units = Percentage inhibition/(1 − Percentage inhibition)

(Ac1 is the absorbance value at 560 nm for blank control 1, 20 μL SOD assay buffer, 160 μL NBT/enzyme working solution, and 20 μL reaction starter working solution; Ac2 is the absorbance value at 560 nm for blank control 2, 40 μL SOD assay buffer and 160 μL NBT/enzyme working solution; As is the absorbance value at 560 nm for each group of samples).

### 4.12. Effect of Caesalmin C on Rpt-3::GFP Expression in C. elegans

Transgenic GR2183 nematodes were transfected with *rpt-3*::GFP fusion protein, and the intensity of green fluorescence was enhanced in nematodes when the compound was able to promote the expression of the proteasome subunit *rpt-3*. Synchronized L1 larvae of GR2183 were received on fresh NGM plates with or without caesalmin C, and treated at 20 °C for 3 days. Each group of nematodes was collected and washed three times with M9 buffer. These nematodes were fixed in the center of the slide with 20 mM sodium azide, observed, and recorded under a fluorescent microscope (DS-Ri2; Nikon, Japan). The fluorescence intensity of each group of nematodes was analyzed by ImageJ software, and the results were expressed as histograms. At least 30 nematodes in each group were subjected to three independent experimental replicates.

### 4.13. Statistical Analysis

Statistical analysis was performed using SPSS 23.0 software. Statistical differences were determined using the one-way ANOVA, with the mean ± SD indicating data, and asterisks were used to indicate significant differences (**** *p* < 0.0001, *** *p* < 0.001, ** *p* < 0.01, * *p* < 0.05, “ns” means no significant difference). The Kruskal-Wallis test was used if the data did not conform to normal distribution. Between-group differences in paralysis data were statistically analyzed using the Kaplan-Meier and log-rank tests for survival analysis in SPSS software. All results were plotted using GraphPad Prism 9.0.

## 5. Conclusions

**Figure 6:**
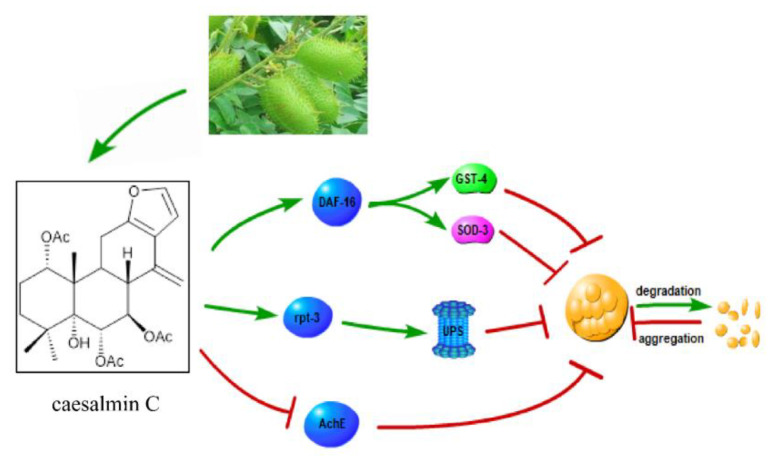
Schematic diagram of caesalmin C resistance to Aβ-induced toxicity in nematodes. The three pathways shown resist Aβ-induced toxicity by promoting the degradation of highly toxic Aβ oligomers or by inhibiting the aggregation of less toxic Aβ into highly toxic oligomers.

In summary, caesalmin C activated partial nuclear translocation of DAF-16 and up-regulated the expressions of detoxification gene *gst-4* and antioxidant gene *sod-3*, thereby strengthening stress resistance. The pro and con results showed that caesalmin C resistance to Aβ-induced toxicity required the involvement of the DAF-16 signaling pathway, but not the SKN-1 and HSF-1 signaling pathways. In addition, caesalmin C may also resist Aβ-induced toxicity by inhibiting AchE activity and activating the ubiquitin-proteasome pathway. The potential signal mechanisms involved are shown in Figure 6.

## Data Availability

The data used to support the findings of this study are available from the corresponding author on reasonable request.

## Supplementary Materials

The following supporting information can be downloaded at: www.mdpi.com/article/10.3390/ijms23126871/s1.
