# Supplementary material for: Diterpenoid Caesalmin C Delays Aβ-Induced Paralysis Symptoms via the DAF-16 Pathway in Caenorhabditis elegans
Source: Int J Mol Sci. 2022 Jun 20;23(12):6871. doi: 10.3390/ijms23126871 (PMC9225120; doi:10.3390/ijms23126871)
Supplement: Supplementary file 1 [file ijms-23-06871-s001.zip › ijms-1753446-supplementary.pdf]

Supplementary Figure S1. Analytical Data of caesalmin C

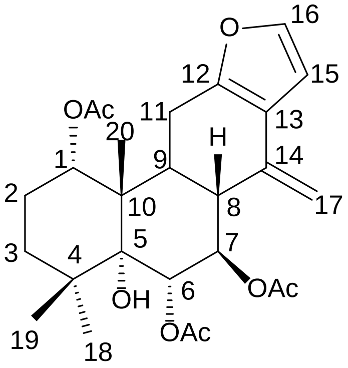

Caesalmin C [1]: $C_{26}H_{34}O_8$, colorless needles. ^{1}H-NMR (400 MHz, $CDCl_3$, δ, ppm, J/Hz): 1.17 (3H, s, H-18), 1.17 (3H, s, H-19), 1.33 (3H, s, H-20), 1.78 (3H, s, 6-OAc), 2.04 (3H, s, 1-OAc), 2.10 (3H, s, 7-OAc), 4.91 (1H, s, H-1), 4.94 (1H, s, H-17a), 5.09 (1H, s, H-17b), 5.60 (2H, s, H-6, 7), 6.41 (1H, s, H-15), 7.23 (1H, s, H-16). ^{13}C-NMR (100 MHz, $CDCl_3$, δ, ppm): 14.0 (C-20), 21.1 (1-OAc), 21.2 (6-OAc), 21.5 (7-OAc), 21.9 (C-2), 23.0 (C-11), 24.8 (C-19), 30.5 (C-18), 32.1 (C-3), 37.8 (C-9), 38.3 (C-4), 41.4 (C-8), 44.3 (C-10), 74.8 (C-1), 75.1 (C-6), 75.3 (C-7), 79.0 (C-5), 105.3 (C-17), 106.3 (C-15), 119.5 (C-13), 138.3 (C-14), 141.8 (C-16), 150.3 (C-12), 168.9 (1-OAc), 170.5 (7-OAc), 170.6 (6-OAc).

Reference

[1] Jiang, R.W., Ma, S.C., But, P.P., Mak, T.C., 2001. New antiviral cassane furanoditerpenes from Caesalpinia minax. J. Nat. Prod. 64, 1266–1272.

Supplementary Figure S2. ¹H NMR (400 MHz) and ¹³C NMR (100 MHz) Spectra of caesalmin C in $CDCl_3$

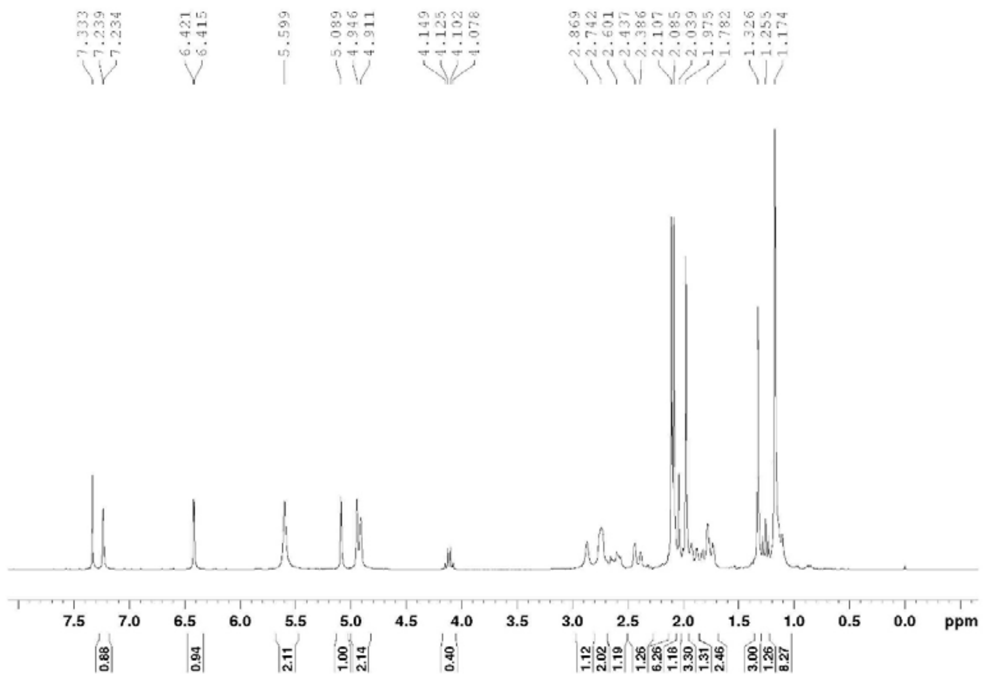

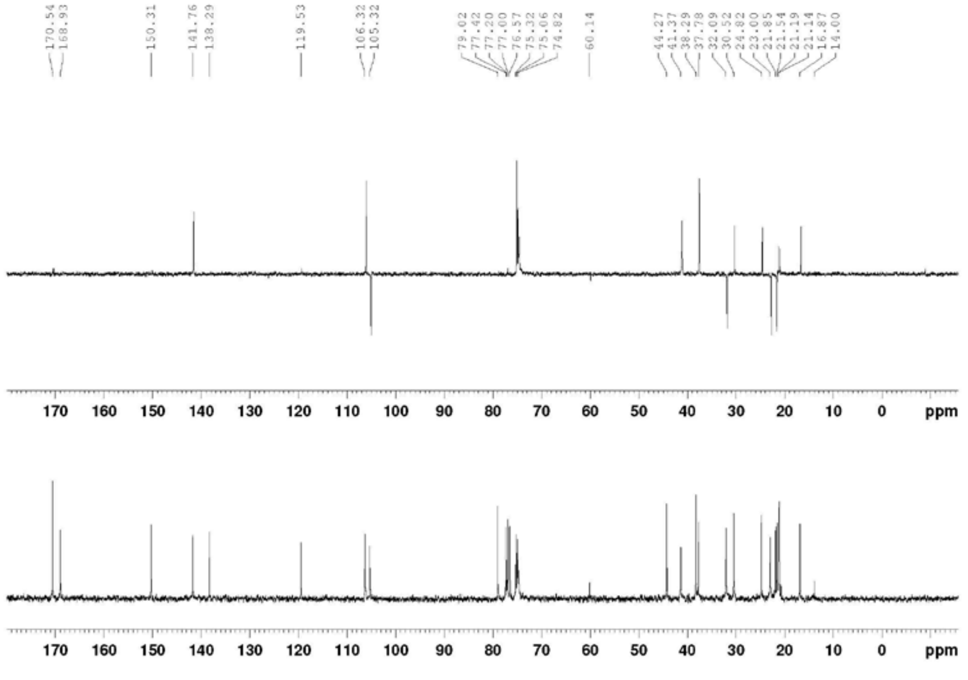

Supplementary Figure S3. The original WB image corresponding to Fig.2. (A) in the manuscript

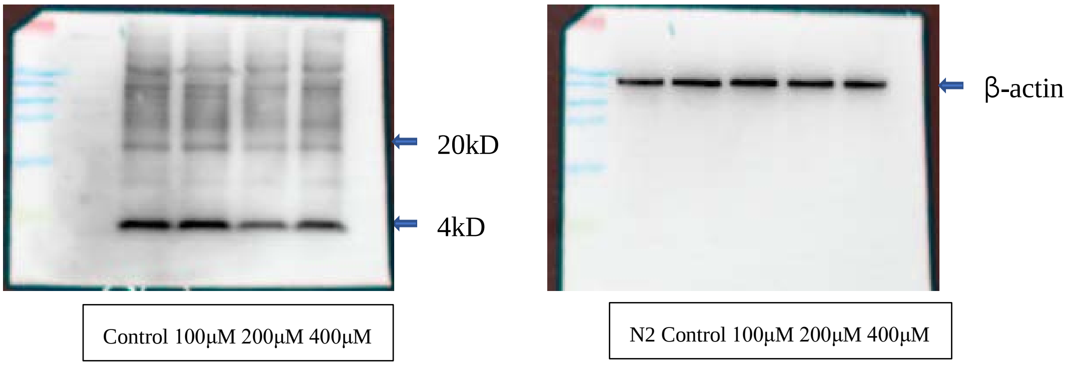

After transferring the protein to the PVDF membrane, the target strip is incubated, exposed, and then closed with membrane regeneration solution before incubating the β-actin strip and exposing it.

Supplementary Figure S4. The original WB image corresponding to Fig.3. (F) in the manuscript.

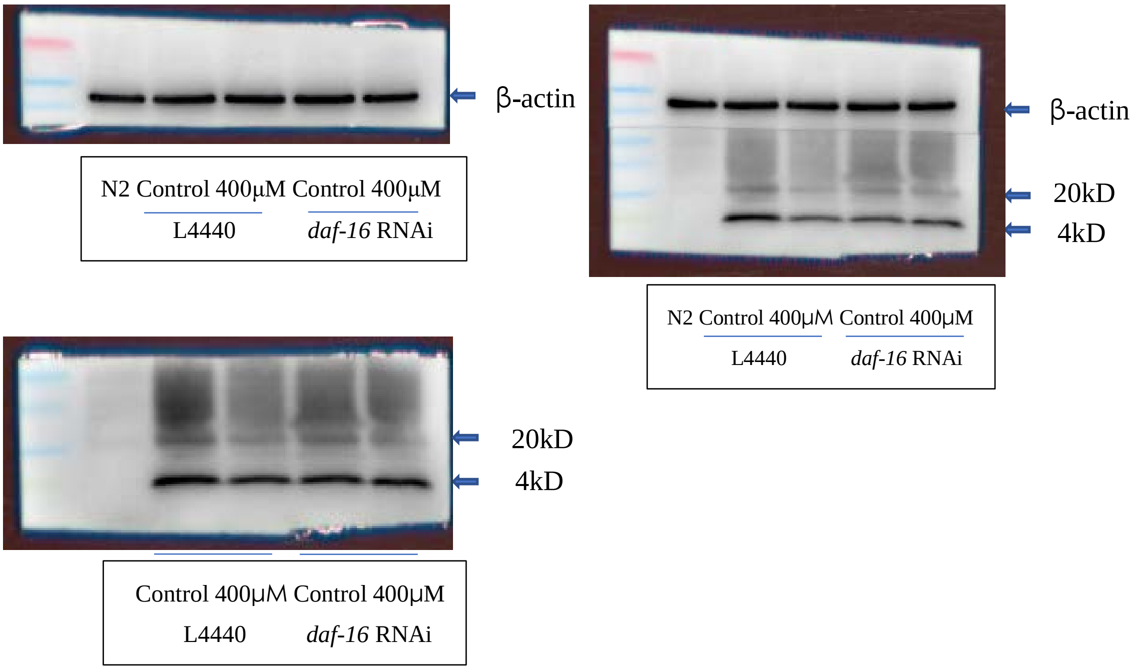

Due to scheduling conflicts in the experiment, after transferring the proteins to the PVDF membrane, they were cut along the location of the β-actin strip and incubated with the corresponding primary antibody for the target strip and the β-actin strip, respectively, and later exposed together. The figure on the right shows the exposure of both strips at the same moment.
